# Systematic reviews: guidance relevant for studies of older people

**DOI:** 10.1093/ageing/afx105

**Published:** 2017-06-24

**Authors:** Susan D. Shenkin, Jennifer K. Harrison, Tim Wilkinson, Richard M. Dodds, John P. A. Ioannidis

**Affiliations:** 1 Geriatric Medicine Unit, University of Edinburgh, Edinburgh, UK; 2 Centre for Cognitive Ageing and Cognitive Epidemiology, University of Edinburgh, UK; 3 Alzheimer Scotland Dementia Research Centre, University of Edinburgh, UK; 4 Centre for Clinical Brain Sciences, University of Edinburgh, Edinburgh, UK; 5 Academic Geriatric Medicine, University of Southampton, Southampton, UK; 6 AGE Research Group, Institute of Neuroscience, Newcastle University, Newcastle, UK; 7 Department of Medicine, Health Research and Policy, Biomedical Data Science, and Statistics, and Meta-Research Innovation Center at Stanford (METRICS), Stanford University, California, USA

**Keywords:** systematic review, meta-analysis, methodology, older people

## Abstract

Systematic reviews and meta-analyses are increasingly common. This article aims to provide guidance for people conducting systematic reviews relevant to the healthcare of older people. An awareness of these issues will also help people reading systematic reviews to determine whether the results will influence their clinical practice. It is essential that systematic reviews are performed by a team which includes the required technical and clinical expertise. Those performing reviews for the first time should ensure they have appropriate training and support. They must be planned and performed in a transparent and methodologically robust way: guidelines are available. The protocol should be written—and if possible published—before starting the review. Geriatricians will be interested in a table of baseline characteristics, which will help to determine if the studied samples or populations are similar to their patients. Reviews of studies of older people should consider how they will manage issues such as different age cut-offs; non-specific presentations; multiple predictors and outcomes; potential biases and confounders. Systematic reviews and meta-analyses may provide evidence to improve older people's care, or determine where new evidence is required. Newer methodologies, such as meta-analyses of individual level data, network meta-analyses and umbrella reviews, and realist synthesis, may improve the reliability and clinical utility of systematic reviews.

## Introduction

Systematic reviews and meta-analyses are increasingly common, with probably more systematic reviews than new randomised trials now published every year [1]. Medline has indexed at least 227,334 articles as ‘systematic reviews’, increasing more than 3-fold in the last decade (from 7,579 in 2006 to 24,517 in 2015). The increase in the number of meta-analyses has been even steeper [1]. The proportion of these systematic reviews specifically indexed in Medline as including participants over age 65 has remained fairly static at around 8% (593 [7.8%] in 2006; 1846 [7.5%] in 2015), but this is likely to be a gross underestimate since many systematic reviews do not get indexed with specific age ranges, and most reviews include individuals regardless of age. Clinical trials in the same period increased less steeply—from 27,125 in 2006 to 34,270 in 2015. The attraction of systematic reviews and meta-analyses is clear: the increasing number and complexity of primary research studies makes it challenging for researchers—or clinicians—to be aware of, assimilate and critically evaluate all the available evidence.

A good systematic review will not only identify the published and unpublished literature on a specific question, but summarise the findings, critically appraise the included studies, and make recommendations on how this should influence clinical practice and future research. It may or may not include a meta-analysis (a statistical summary of the evidence). This is a key step in reducing research waste (http://blogs.bmj.com/bmj/2015/10/29/how-systematic-reviews-can-reduce-waste-in-research). However, many published reviews and meta-analyses are redundant, misleading or serve conflicting interests [1], and may compound the limitations of primary studies, rather than critically exposing them [2]. This article aims to provide guidance for people conducting systematic reviews relevant to the healthcare of older people, while acknowledging that there is no universal definition of ‘older person’. An awareness of these issues will also help people reading systematic reviews to determine whether the results will influence their clinical practice.

## Guidance on conducting a systematic review

### Protocols

Before performing a systematic review, the reviewer should first ensure that the question has not already been answered, and check whether a review is already ongoing elsewhere. To check for published reviews, one can search databases which collate systematic reviews and meta-analyses (e.g. https://discover.dc.nihr.ac.uk/portal/search/signals), the Cochrane Library (http://www.cochranelibrary.com/) or bibliographic databases such as Medline, PsycInfo or CINAHL. Databases e.g. PROSPERO (https://www.crd.york.ac.uk/PROSPERO/) are available to register a planned review. PROSPERO is an open access resource that does not provide critical review of protocols, but provides a searchable repository to check if this review is already complete, ongoing or planned. Once the new protocol is finalised, it would be useful to register it if possible. PROSPERO only allows registration of topics related to health outcomes. Other databases e.g. http://www.researchregistry.com/ allow registration of systematic reviews on any topic, at any stage in the review process. The protocol can be updated later to make clear any subsequent changes.

### Practical advice

**Figure 1.**
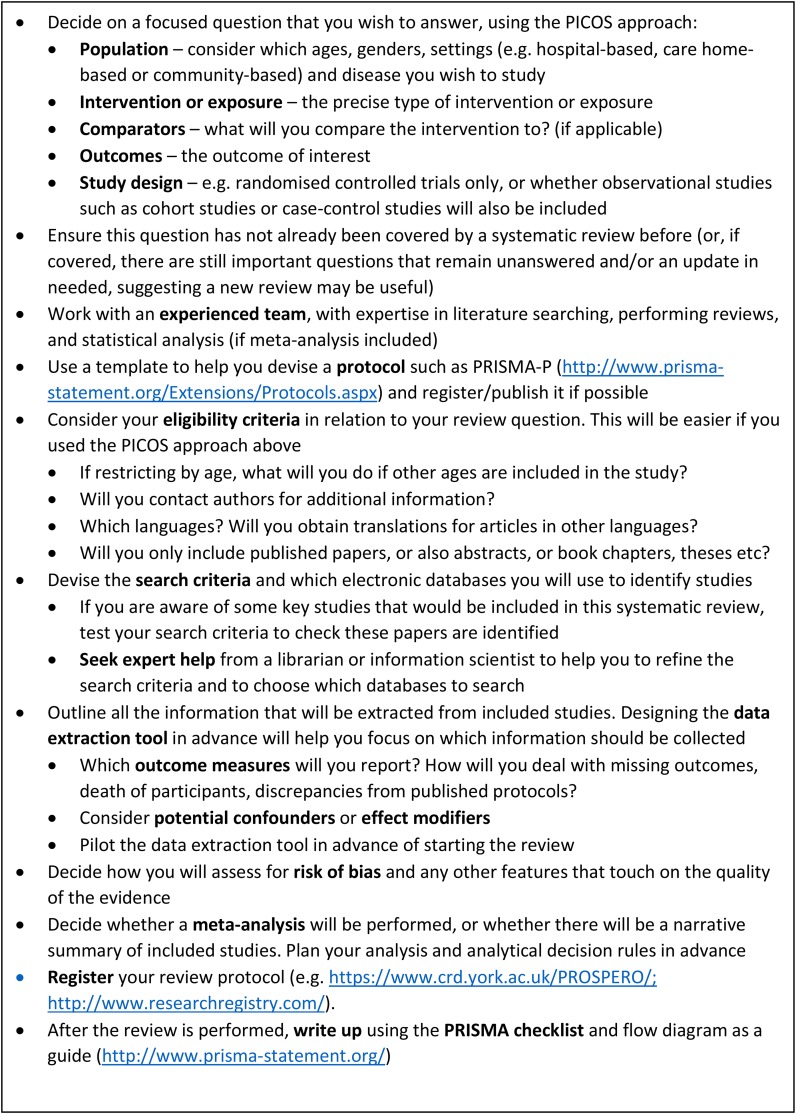
Good practice points for performing systematic reviews and meta-analyses in ageing research.

Detailed guidance on performing systematic reviews is available elsewhere (https://www.york.ac.uk/crd/guidance/). We have suggested some good practice points (Figure 1). The PRISMA-P [3] and PRISMA [4] statements provide guidance for designing and reporting systematic reviews, respectively. A checklist is also available of what should be included. The Cochrane Handbook of systematic reviews of interventions [5] provides comprehensive guidance on all aspects of systematic reviews and meta-analysis, and online modules are freely available from Cochrane Training (http://training.cochrane.org). This advice is most relevant for randomised controlled trials (RCTs) of interventions in healthcare, e.g. pharmacological treatments such as thrombolysis in acute ischaemic stroke [6] or complex interventions such as multi-component interventions to prevent delirium [7].

However, much research relevant to older people is not in the form of randomised controlled trials: this may be due to the difficultly of performing RCTs for the questions of interest to specialists in the care of older people, encompassing complex multidisciplinary, multi-component interventions (e.g. stroke units) where it is not easy to tease out the individual components. The population of interest typically includes frail older people with multiple medical conditions and ranges of functional impairment, thus there is very large heterogeneity, and the restricted eligibility criteria of RCTs may leave out many types of patients and settings.

Where RCTs are not possible, feasible or ethical, observational study designs (cohort or case–control studies) may provide some evidence for healthcare interventions. For a discussion, with case examples, of how observational studies may provide moderate to (rarely) high strength evidence in systematic reviews of healthcare interventions see [8]. Observational methods are also a main method for exploring associations between harmful risk-factors and outcomes, e.g. the association between obesity and dementia [9], and there are specific aspects that should be considered [10].

Whether a systematic review is of trials, or observational studies, the assessment of risk of bias of each included study, and of the review overall is key. Authors should ensure that they do not ‘sanctify results from poor studies’ [2] and include clear critical appraisal of included data. For specific guidance on conducting systematic reviews of observational studies see online step by step guidance (http://www.ccace.ed.ac.uk/research/software-resources/systematic-reviews-and-meta-analyses) and guidance for early career researchers in epidemiology [11]. The MOOSE guidelines [12] provide a standard for systematic reviews of observational studies, but have not been updated since 2000. The STROBE statement (https://www.strobe-statement.org), outlines the issues that should be considered in the reporting of epidemiology studies. Software is available (e.g. Covidence https://www.covidence.org/, EPPI-Reviewer, http://community.cochrane.org/tools/review-production-tools/eppi-reviewer/about or Rayyan—https://rayyan.qcri.org/) which keeps all data secure remotely, and allows collaboration between authors in different locations: this can facilitate independent assessment, allow accountability and is a method to keep track of the process. It is not a substitute for adequate training in review methodology and statistical literacy.

## Issues to consider in studies of older people

Geriatricians are well aware of the complexity of dealing with older patients, and the paucity of evidence to guide their practice. When performing a systematic review relevant to the healthcare of older people, there are some specific issues that should be considered.

### Age

There is no generally agreed criterion for definition of ‘older people’. A cut-off of over 60 or 65 years is often used (http://www.who.int/healthinfo/survey/ageingdefnolder/en/). However, in the developed world, the syndromes most relevant to geriatricians (e.g. dementia, frailty) are most common after age 80. In other areas, such as sub-Saharan Africa, ‘older people’ may be defined as over age 50 (http://www.who.int/healthinfo/survey/ageingdefnolder/en/index.html). It is important to be clear which studies will be eligible for inclusion: those with participants at all ages, or only those which include older people. Reviewers should predetermine how they will deal with studies, particularly randomised controlled trials, which have different recruitment strategies relating to age. Many studies exclude patients over a certain age (e.g. 75 years), in which case geriatricians will be wary about extrapolating the results to their clinical practice. If there is no age restriction, can results be examined specifically for older people (e.g. >65; 75; 85 years)? Even if older people are recruited, is there evidence of selection bias, i.e. are included patients different from those in routine clinical practice? Research studies generally recruit people who are healthier, better educated and of higher (less deprived) socioeconomic status. It is important to include a baseline table of participant characteristics so that readers can determine if the results are directly relevant to their own patients or extrapolations need to be made. Many studies will not present the overall age range, with mean and SD, but instead split the sample into age decades, or other categories. This can make it difficult to compare findings between studies—and authors of systematic reviews should attempt to obtain original information from study authors. We would encourage authors of individual studies to include overall age range, mean and SD as well as reporting in categories, and to make the individual-participant data available for reuse as soon as possible. For reviews, as a minimum, mean or median age (in years) and a description of the distribution (standard deviation, range or inter-quartile range) is required. If there is a wide age range, then it is helpful to report the numbers and proportion over 65 years. Also, reviewers should consider providing information on the numbers of the oldest participants, e.g. numbers and proportion of those aged over 80 or 85 years.

### Non-specific presentation of disease

Systematic reviews can be challenging when there is lack of agreement on what to include as predictor or outcome variables, or the definition of participants. For example, there is variability in what is meant by frailty [13], or ‘care home resident’ [14]. Many studies will focus on a single predictor or outcome. It is important to consider if this outcome is relevant, or a ‘surrogate’ which may, or may not relate to clinical outcomes, e.g. body sway, rather than number of falls. In addition, although one outcome may be reported, it is important to consider other potentially relevant outcomes: e.g. treatments for incontinence will report the impact on continence, but may not report whether there were also impacts on rate of falls or cognition. It is also important to consider how a review will deal with outcomes which are reported inconsistently across trials (e.g. different cognitive tests or functional assessments). How will missing outcome data be handled? Is it missing at random, or will missing data influence the result? Can additional information be obtained from the primary investigators on outcomes that are not reported? Consider contacting the corresponding author from the original paper, or any author whose current contact details can be found. What impact does death of participants have on the results? It is also important, particularly in clinical trials, to determine whether the outcomes are those that were planned in the original protocol [2].

### Inclusion/exclusion criteria

Are the people included similar to routine clinical practice? For example, are people excluded on the basis of age, or co-morbidities that are common in older people? Be aware of international variation in healthcare practices: the infrastructure in a residential home, or support by families, varies widely internationally. There is also international variation in attitudes to ageing by older people themselves, and others [15] which may affect the impact of interventions. Can the findings be extrapolated to a clinical population? If data on populations of interest (e.g. age over 90; care home residents; people lacking capacity) are lacking, identify and discuss this gap in the literature.

### Study design

Be clear if the studies included are intervention studies (e.g. randomised controlled trials) or observational studies (e.g. prospective or retrospective cohort studies, or case–control studies), and what methodological considerations are important for each. Note that observational designs identify associations, but cannot prove causation: e.g. the association between diabetes and risk of falls may be due to complications of diabetes, or hypoglycaemia, or unmeasured confounders such as hypotension or other unknown or convoluted reasons [16].

### Confounding

Particularly for observational studies, it is important to consider if the results could be explained by another variable that may be unobserved. Confounding variables may make it appear—incorrectly—that an observed exposure or predictor is (or is not) causally associated with an outcome. In studies of older people, potentially important confounders, particularly from earlier life, should be considered, such as socioeconomic status. For example, socioeconomic status probably explains the observed association between caffeine intake and IQ [17].

### Effect modification

This occurs in studies where an exposure has a different effect in different subgroups: effect modifiers are associated with the outcome, not the exposure. For example, older (or frailer, or cognitively impaired) people may respond differently to an intervention than younger (or fitter, or cognitively normal) people.

### Bias

Bias is an incorrect estimation of the association, e.g. between exposure and outcome, due to a systematic error. The assessment of bias is a critical component of systematic reviews of RCTs and observational studies, and is the main component of what has often been termed ‘quality assessment’. Careful consideration should be given in advance as to how studies will be assessed (see [14] for some guidance relevant to studies of older people: including the Cochrane Risk of Bias tool [19] for RCTs, ROBANS [18] for observational studies). Summary scores are generally discouraged as they can mask complexity and give equal weight to aspects of quality assessment that could have minimal, or large, impact on the applicability of results. Note, however, that there can be considerable inter-individual variation in risk of bias assessments (e.g. [19]).

For all studies including older people, reviewers should consider selection bias—that results will be distorted by including people in the study that are not representative of patients that are looked after in clinical practice (e.g. the hypertension study SPRINT which excluded people aged ≥75 with co-morbidities such as diabetes, heart failure, stroke, dementia [20])—and survival bias—that only those who survive are included in the results, which may give erroneous conclusions about efficacy of treatments or risk-factors for outcomes (e.g. better function appeared to predict care home admission in this study as those with poorer function died before discharge [21]).

### Meta-analysis

A statistical synthesis of the results [22] is not required for a systematic review (either of RCTs or observational data), but this should be performed where possible, while being aware of how to interpret the results in the presence of heterogeneity. It is good practice to contact authors of the original studies to obtain additional data (e.g. if data have been presented in different age bands in different studies): this can be variably successful. It is important to have sufficient statistical expertise in the systematic review team, and to combine this with clinical knowledge to assess if statistical synthesis is appropriate and how it should be interpreted. A summary effect can be tantalisingly succinct, but this should not dissuade authors from critically discussing the limitations of the included studies and, therefore, the summary evidence. Different meta-analyses of the same question can come to different conclusions due to inclusion of studies with diverse protocols and methods.

If presenting data in a forest plot, check that the axes are clearly labelled, so that the size of the effect, and its potential clinical significance, can be gauged. Include confidence intervals of the summary estimate. Is a fixed-effect or random-effect model planned, or will a prediction interval be presented: this provides a predicted range for the true treatment effect in an individual study [19]? Reviews often quote formal measures of heterogeneity such as the *I*^2^ statistic, but it is also important to carefully scrutinise the table of baseline characteristics to see if there is relevant heterogeneity between the individual studies included. Whether meta-analysis is possible or not, systematic reviews should narratively summarise and critically appraise the results across all included studies.

## Using systematic reviews to inform clinical practice and service redesign

It is typically discouraged for systematic reviewers themselves to make *ex officio* guideline-level recommendations. Usually, systematic reviews end with the critical appraisal and synthesis of the evidence. However, these systematic reviews are used eventually by multiple users, ranging from clinical practitioners and readers to guideline committees to inform clinical practice and for service redesign. Systematic reviews have been helpful to prompt changes in clinical practice, when change has not followed publication of individual trials (e.g. thrombolysis for acute ischaemic stroke). Reviews can also be used to support business cases for service redesign (e.g. comprehensive geriatric assessment) or to identify gaps in evidence where research and clinical change is required (e.g. music therapy in the acute hospital). When assessing the relevance of the review to the care of older people, readers should consider the quality of the systematic review itself as well as the studies that are included in it. Are included subjects relevant to clinical practice? Is the intervention feasible in routine care? Tools such as AMSTAR [23] suggest items which should be considered in determining quality of a published systematic review.

## The future of systematic reviews and meta-analysis

Given the limitations of published reviews and meta-analyses, it is essential that clinicians treat the results with appropriate scepticism, and make informed choices for any proposed change in practice. Although systematic reviews and meta-analyses may be seen as the ‘top’ of the evidence pyramid, this only applies if the reviews are performed to the highest standard. The quality of reporting of published reviews has improved since the publication of the PRISMA statement [4], but the large majority of reviews and meta-analyses continue to be flawed, redundant, misleading or clinically not useful [1]. The requirement that authors disclose any conflict of interest is important: results and conclusions are often biased when authored by company employees, or if a study is industry sponsored [24]. Crucially, there is an increasing call from funders as well as researchers that all clinical trials (and observational studies) are reported (e.g. www.AllTrials.net) and that individual level data is made available as soon as possible. This requires additional infrastructure and funding, especially for studies that require significant storage space such as imaging data (e.g. Alzheimer's Disease Neuroimaging Initiative http://adni.loni.usc.edu/).

There are several methodologies which may improve the reliability and clinical utility of systematic reviews [1], and these are discussed here.

### Individual patient meta-analysis

Where the data from individual studies are combined (while retaining the clustering of patients within their individual study)—has become increasingly common [25]. It is often limited by the provision of original data, and may result in analysis of only a subset of otherwise eligible studies, and thus can be prone to reporting bias. The meta-analysis of data across multiple observational studies can allow a summary estimate of risk-factors, e.g. the effect of smoking and smoking cessation on cardiovascular mortality in older people [26], but is still at risk of residual confounding. Selection for inclusion of studies based on already known results can be a problem in meta-analyses of individual level data [27].

### Network meta-analysis

Network meta-analysis allows multiple treatments to be compared using direct comparisons within randomised controlled trials and indirect comparisons using a common comparator [28]. This allows both direct and indirect evidence to be used to synthesise the published evidence, such as the effectiveness and adverse effects of antidepressants [29]. Treatment networks can quantitatively analyse data for all treatment comparisons for the same disease. Multiple treatments meta-analysis can rank the effectiveness of many treatments in a network. However, for this to add useful information to simpler methods of data synthesis, large amounts of data are required, and care must be taken in interpretation of the direct and indirect comparisons, which can sometimes disagree.

### Prospective meta-analysis

Trials can be designed with the explicit predefined purpose of future combination in a meta-analysis [2]: this approach can combine studies with different designs, interventions, comparisons, setting and populations. It is ideal for diverse interventions such as falls prevention programmes [30], but is uncommon as they require significant collaboration, co-ordination and advance planning.

*Umbrella reviews* [2] are systematic reviews of systematic reviews: they may summarise all systematic reviews or meta-analyses performed on a given topic, e.g. all treatments for a condition or set of conditions; or all risk-factors assessed for some disease (e.g. Alzheimer's disease, [31]); or all associations that a specific risk factor has been evaluated for in relationship to a variety of outcomes/diseases (e.g. [32]). These reviews may permit understanding of the amount and credibility of the evidence in a large field, knowledge gaps and the main sources of heterogeneity and bias.

*Rapid reviews* [33] are gaining popularity as methods to make reviews and subsequent treatment decisions more rapidly than allowed by full systematic reviews. If appropriately supervised they may be useful for training, and may markedly speed up the review process, but as yet there is no consensus on definition or methodology. Similarly, participation in adjudication of studies for systematic reviews as in Cochrane Crowd (http://crowd.cochrane.org) can provide some experience and training. Cochrane crowd are not a substitute for a systematic review by a team with appropriate expertise.

### Realist synthesis

Realist synthesis has been suggested as a pragmatic approach for evaluating complex interventions [34] e.g. models of care for older people living in care homes [35]. It tries to establish, ‘what works for whom in what circumstances and in what respects?’ [36], rather than looking at single interventions. However, the lack of predefined questions/outcomes risks focussing on positive studies, and it may be most useful as an exploratory method for complex and multidisciplinary interventions.

## Conclusions

Despite their limitations, systematic reviews are useful for practising geriatricians to inform clinical care, and to identify where further research is needed. It is essential that any review, either using traditional or more novel methodologies, is done to a high standard, considering both general issues for good quality reporting, but also paying particular attention to specific issues relevant to the health of older people.

**Key points**

- Systematic reviews must be planned and performed in a transparent and methodologically robust way: guidelines are available, and a carefully designed protocol will help.
- A table of baseline characteristics will help practising geriatricians in relating the studied populations to their patients.
- Geriatricians should consider how reviews deal with: different age cut-offs; non-specific presentations; multiple predictors and outcomes; potential biases and confounders.
- Reviews may determine where new evidence is required to improve the care of older people.
- Meta-analyses of individual level data, network meta-analyses and umbrella reviews offer interesting possibilities for more in-depth or broader views of the evidence.

## Supplementary Material

Supplementary Data
