# Systematic reviews: guidance relevant for studies of older people

**DOI:** 10.1093/ageing/afx185

**Published:** 2017-12-02

**Authors:** Susan D Shenkin, Jennifer K Harrison, Tim Wilkinson, Richard M Dodds, John P A Ioannidis

Due to a typographical error, the original version of the above article read ‘Cochrane crowd are a substitute for a systematic review by a team with appropriate expertise’. This has now been corrected online to read ‘Cochrane crowd are not a substitute for a systematic review by a team with appropriate expertise’. The authors apologize for this error.

