# Supplementary material for: Systematic reviews: guidance relevant for studies of older people
Source: Age Ageing. 2017 Jun 24;46(5):722–8. doi: 10.1093/ageing/afx105 (PMC5860219; doi:10.1093/ageing/afx105)
Supplement: Supplementary Data [file 17-0482_sd.docx]

# Systematic reviews: guidance relevant for studies of older people

**Susan D Shenkin^1,2^, Jennifer K Harrison^1,2,3^, Tim Wilkinson^4^, Richard M Dodds^5,6^, John PA Ioannidis^7^**

**Appendix: full list of references**

1. Ioannidis JP. The Mass Production of Redundant, Misleading, and Conflicted Systematic Reviews and Meta-analyses. Milbank Q. 2016 9/2016;94(3):485-514.

2. Ioannidis J. Next-generation systematic reviews: prospective meta-analysis, individual-level data, networks and umbrella reviews. Br J Sports Med. 2017 2/21/2017.

3. Moher D, Shamseer L, Clarke M, Ghersi D, Liberati A, Petticrew M, et al. Preferred reporting items for systematic review and meta-analysis protocols (PRISMA-P) 2015 statement. Syst Rev. 2015 1/1/2015;4:1.

4. Moher D, Liberati A, Tetzlaff J, Altman DG. Preferred reporting items for systematic reviews and meta-analyses: the PRISMA statement. Ann Intern Med. 2009 8/18/2009;151(4):264-9, W64.

5. Collaboration TC. Cochrane handbook for systematic reviews of interventions Version 5.1.0. JPT H, S G, editors2011.

6. Wardlaw JM, Murray V, Berge E, del Zoppo GJ. Thrombolysis for acute ischaemic stroke. Cochrane Database Syst Rev. 2014 7/29/2014(7):CD000213.

7. Siddiqi N, Harrison JK, Clegg A, Teale EA, Young J, Taylor J, et al. Interventions for preventing delirium in hospitalised non-ICU patients. Cochrane Database of Systematic Reviews. 2016 2016(3):1465-858.

8. O'Neil M, Berkman N, Hartling L, Chang S, Anderson J, Motu'apuaka M, et al. Observational evidence and strength of evidence domains: case examples. Syst Rev. 2014 4/23/2014;3:35.

9. Pedditizi E, Peters R, Beckett N. The risk of overweight/obesity in mid-life and late life for the development of dementia: a systematic review and meta-analysis of longitudinal studies. Age Ageing. 2016 1/2016;45(1):14-21.

10. Egger M, Davey Smith G, Schneider M. Systematic reviews of observational studies. In: Egger M, Davey Smith G, Altman D, editors. Systematic Reviews in Healthcare: meta-analysis in context: WIley; 2008. p. 211-27.

11. Denison HJ, Dodds RM, Ntani G, Cooper R, Cooper C, Sayer AA, et al. How to get started with a systematic review in epidemiology: an introductory guide for early career researchers. Arch Public Health. 2013 8/7/2013;71(1):21.

12. Stroup DF, Berlin JA, Morton SC, Olkin I, Williamson GD, Rennie D, et al. Meta-analysis of observational studies in epidemiology: a proposal for reporting. Meta-analysis Of Observational Studies in Epidemiology (MOOSE) group. JAMA. 2000 4/19/2000;283(15):2008-12.

13. A A, C H, A V, ASV S, SJ C, AMJ M, et al. The relationship between preoperative frailty and outcomes following Transcatheter Aortic Valve Implantation (TAVI): *a systematic review and meta-analysis*. Eur Heart J Qual Care Clin Outcomes. 2016 2016.

14. Harrison JK, Reid J, Quinn TJ, Shenkin SD. Using quality assessment tools to critically appraise ageing research: a guide for clinicians. Age Ageing. 2016 12/7/2016.

15. Laidlaw K, Power MJ, Schmidt S. The Attitudes to Ageing Questionnaire (AAQ): development and psychometric properties. Int J Geriatr Psychiatry. 2007 4/2007;22(4):367-79.

16. Yang Y, Hu X, Zhang Q, Zou R. Diabetes mellitus and risk of falls in older adults: a systematic review and meta-analysis. Age Ageing. 2016 11/2016;45(6):761-7.

17. Corley J, Jia X, Kyle JA, Gow AJ, Brett CE, Starr JM, et al. Caffeine consumption and cognitive function at age 70: the Lothian Birth Cohort 1936 study. Psychosom Med. 2010 2/2010;72(2):206-14.

18. Kim SY, Park JE, Lee YJ, Seo HJ, Sheen SS, Hahn S, et al. Testing a tool for assessing the risk of bias for nonrandomized studies showed moderate reliability and promising validity. J Clin Epidemiol. 2013 4/2013;66(4):408-14.

19. Higgins JP, Altman DG, Gotzsche PC, Juni P, Moher D, Oxman AD, et al. The Cochrane Collaboration's tool for assessing risk of bias in randomised trials. BMJ. 2011 10/18/2011;343:d5928.

20. Williamson JD, Supiano MA, Applegate WB, Berlowitz DR, Campbell RC, Chertow GM, et al. Intensive vs Standard Blood Pressure Control and Cardiovascular Disease Outcomes in Adults Aged >/=75 Years: A Randomized Clinical Trial. JAMA. 2016 6/28/2016;315(24):2673-82.

21. Astell AJ, Clark SA, Hartley NT. Predictors of discharge destination for 234 patients admitted to a combined geriatric medicine/old age psychiatry unit. Int J Geriatr Psychiatry. 2008 9/2008;23(9):903-8.

22. Borenstein M, Hedges LV, Higgins JPT, Rothstein HR. Introduction to Meta-Analysis: John Wiley & Sons; 2009.

23. Shea BJ, Grimshaw JM, Wells GA, Boers M, Andersson N, Hamel C, et al. Development of AMSTAR: a measurement tool to assess the methodological quality of systematic reviews. BMC Med Res Methodol. 2007 2/15/2007;7:10.

24. Ebrahim S, Bance S, Athale A, Malachowski C, Ioannidis JP. Meta-analyses with industry involvement are massively published and report no caveats for antidepressants. J Clin Epidemiol. 2016 2/2016;70:155-63.

25. Riley RD, Lambert PC, Abo-Zaid G. Meta-analysis of individual participant data: rationale, conduct, and reporting. BMJ. 2010 2/5/2010;340:c221.

26. Mons U, Muezzinler A, Gellert C, Schottker B, Abnet CC, Bobak M, et al. Impact of smoking and smoking cessation on cardiovascular events and mortality among older adults: meta-analysis of individual participant data from prospective cohort studies of the CHANCES consortium. BMJ. 2015 4/20/2015;350:h1551.

27. Flegal KM, Ioannidis JPA. A meta-analysis but not a systematic review: an evaluation of the Global BMI Mortality Collaboration. J Clin Epidemiol. 2017 4/20/2017.

28. Mills EJ, Thorlund K, Ioannidis JP. Demystifying trial networks and network meta-analysis. BMJ. 2013 5/14/2013;346:f2914.

29. Thorlund K, Druyts E, Wu P, Balijepalli C, Keohane D, Mills E. Comparative efficacy and safety of selective serotonin reuptake inhibitors and serotonin-norepinephrine reuptake inhibitors in older adults: a network meta-analysis. J Am Geriatr Soc. 2015 5/2015;63(5):1002-9.

30. Province MA, Hadley EC, Hornbrook MC, Lipsitz LA, Miller JP, Mulrow CD, et al. The effects of exercise on falls in elderly patients. A preplanned meta-analysis of the FICSIT Trials. Frailty and Injuries: Cooperative Studies of Intervention Techniques. JAMA. 1995 5/3/1995;273(17):1341-7.

31. Bellou V, Belbasis L, Tzoulaki I, Middleton LT, Ioannidis JP, Evangelou E. Systematic evaluation of the associations between environmental risk factors and dementia: An umbrella review of systematic reviews and meta-analyses. Alzheimers Dement. 2017 4/2017;13(4):406-18.

32. Theodoratou E, Tzoulaki I, Zgaga L, Ioannidis JP. Vitamin D and multiple health outcomes: umbrella review of systematic reviews and meta-analyses of observational studies and randomised trials. BMJ. 2014 4/1/2014;348:g2035.

33. Haby MM, Chapman E, Clark R, Barreto J, Reveiz L, Lavis JN. What are the best methodologies for rapid reviews of the research evidence for evidence-informed decision making in health policy and practice: a rapid review. Health Res Policy Syst. 2016 11/25/2016;14(1):83.

34. Rycroft-Malone J, McCormack B, Hutchinson AM, DeCorby K, Bucknall TK, Kent B, et al. Realist synthesis: illustrating the method for implementation research. Implement Sci. 2012 4/19/2012;7:33.

35. Goodman C, Dening T, Gordon AL, Davies SL, Meyer J, Martin FC, et al. Effective health care for older people living and dying in care homes: a realist review. BMC Health Serv Res. 2016 7/16/2016;16:269.

36. Pawson R, Greenhalgh T, Harvey G, Walshe K. Realist review--a new method of systematic review designed for complex policy interventions. J Health Serv Res Policy. 2005 7/2005;10 Suppl 1:21-34.
